# Association Between Rehabilitation Frequency and Functional Outcomes After Burn Injury: A Single-Center Retrospective Analysis of Confounding by Indication

**DOI:** 10.3390/ebj7010006

**Published:** 2026-01-19

**Authors:** Yazeed Temraz, Theeb Al Salem, Shaimaa Khan, Raghad Alshehri, Lina Alosaimi, Mariam Hantoul, Rahaf Alrajhi, Rayya Alabdali, Amal Bahumayim, Ibtihal Al Jafin, Fai Al Qazlan, Abdulmajeed Al Ehaideb

**Affiliations:** 1 Rehabilitation Department, Ministry of the National Guard-Health Affairs, Riyadh 11426, Saudi Arabia; alsalemth@mngha.med.sa (T.A.S.); khansh@mngha.med.sa (S.K.); alshehrira6@mngha.med.sa (R.A.); alosaimili@mngha.med.sa (L.A.); hantoulma@mngha.med.sa (M.H.); alrajhira1@mngha.med.sa (R.A.); alabdalira@mngha.med.sa (R.A.); bahumayimam@mngha.med.sa (A.B.); aljafinib@mngha.med.sa (I.A.J.); alqazlanfa@mngha.med.sa (F.A.Q.); alehaidebab1@mngha.med.sa (A.A.E.); 2 King Abdullah International Medical Research Center (KAIMRC), Riyadh 11426, Saudi Arabia

**Keywords:** burn rehabilitation, mixed-depth burns, scar outcome, confounding by indication, physical therapy, occupational therapy, Vancouver Scar Scale, functional independence measure

## Abstract

Objective: To identify key predictors of clinical outcomes in burn survivors and clarify the role of mixed-depth burns and confounding by indication in observational rehabilitation research. Design: Retrospective cohort study using data from a burn rehabilitation registry (January 2024 to July 2025). Setting: Burn rehabilitation center. Participants: 120 adult patients (age ≥ 18 years) with burns affecting ≥1% total body surface area (TBSA) and complete baseline data. Interventions: Not applicable. Main Outcome Measures: Primary outcome was functional improvement (ΔFIM). Secondary outcomes included pain reduction (ΔPain), scar severity (Vancouver Scar Scale; VSS), Activities of Daily Living (ADL) improvement, and Range of Motion (ROM) recovery. Multivariable linear and logistic regression models were used to identify predictors. Results: Patients achieved significant improvements in function (mean ΔFIM = 11.3 ± 8.9 points) and pain (mean ΔPain = 1.28 ± 0.81). Having a mixed-depth burn was the strongest predictor of worse scar outcomes (β = 2.52, 95% CI: 0.93 to 4.12, *p* = 0.002) and failure to achieve full ROM (OR = 0.089, 95% CI: 0.008 to 0.930, *p* = 0.043). An apparent association between inpatient ward care and better scar outcomes (β = −1.30, *p* = 0.020) was determined to be an artifact of confounding by indication, as the outpatient group had a higher proportion of high-risk mixed-depth burns (6.2% vs. 3.5%). Longer therapy duration was the only significant predictor of achieving ADL goals (OR = 1.014, 95% CI: 1.002 to 1.026, *p* = 0.025). Conclusions: Injury characteristics, particularly the presence of a mixed-depth burn, emerged as the dominant predictors of long-term scar and functional outcomes. This study identifies mixed-depth burns as a potentially high-risk clinical phenotype requiring targeted therapeutic strategies and demonstrates the critical importance of accounting for confounding by indication when evaluating rehabilitation outcomes in observational burn research.

## 1. Introduction

Severe burn injuries are among the most complex forms of trauma, leading to long-term disability, disfigurement and reduced quality of life [1,2,3]. Rehabilitation is a cornerstone of modern burn care, delivered through multidisciplinary programs that include physical therapy, occupational therapy, splinting and scar management [4,5,6]. Its main goals are to preserve range of motion (ROM), maximize functional independence and support scar maturation, participation and psychosocial recovery [2,7].

The trajectory of recovery is largely determined by the characteristics of the initial injury. Greater total body surface area (TBSA), deeper burns and involvement of major joints are consistently associated with more complications, pathological scarring and joint contracture [1,2,8,9,10,11]. Accordingly, guidelines and expert reviews recommend that the content and amount of rehabilitation be scaled to injury severity and joints at risk for contracture, with early mobilization and continued therapy across Intensive Care Unit (ICU), ward and outpatient settings [4,5,12].

Despite this, the evidence base for specific rehabilitation “doses” in adult burn care remains limited. Most data derive from heterogeneous observational cohorts and small trials, with substantial variation in timing, frequency and content of interventions [6,13,14,15]. In routine practice, patients with more extensive or deeper burns appropriately receive more intensive and prolonged rehabilitation, so therapy volume is closely linked to injury severity and prognosis. This confounding by indication can obscure true dose–response relationships and makes it difficult to separate the effect of rehabilitation from the dominant influence of the underlying injury in registry analyses [6,8].

Burn classification adds further complexity. Clinical labels such as superficial, partial thickness and full thickness remain important, but individual wounds often contain areas of different depth within the same anatomical region [2,11]. Deep dermal and full-thickness components, delayed healing and joint involvement are key risk factors for hypertrophic scarring and contracture [9,10], suggesting that “mixed-depth” burns may represent a distinct high-risk phenotype. Yet such patterns are rarely analyzed as a separate category, and it is unclear whether they are associated with poorer outcomes or require different therapeutic strategies.

Therefore, this study analyzed a real-world cohort of adult burn survivors to address these gaps. The aims were: (1) to describe rehabilitation services and clinical outcomes in a contemporary burn center; (2) to identify key predictors of functional improvement, pain reduction, scar quality and ROM recovery using multivariable regression models; and (3) to specifically examine the impact of mixed-depth burn patterns and clarify the relationship between care setting, therapy dose and patient outcomes, with particular attention to confounding by indication.

## 2. Methods

### 2.1. Study Design and Reporting

This was a retrospective cohort study using a clinical burn rehabilitation registry from a single specialized tertiary hospital center. Reporting followed STROBE and RECORD guidelines. Consistent with the Declaration of Helsinki, participant consent was not required for this retrospective review of anonymized clinical records, following ethics committee approval (No: 00000169025)

### 2.2. Participants

**Figure 1:**
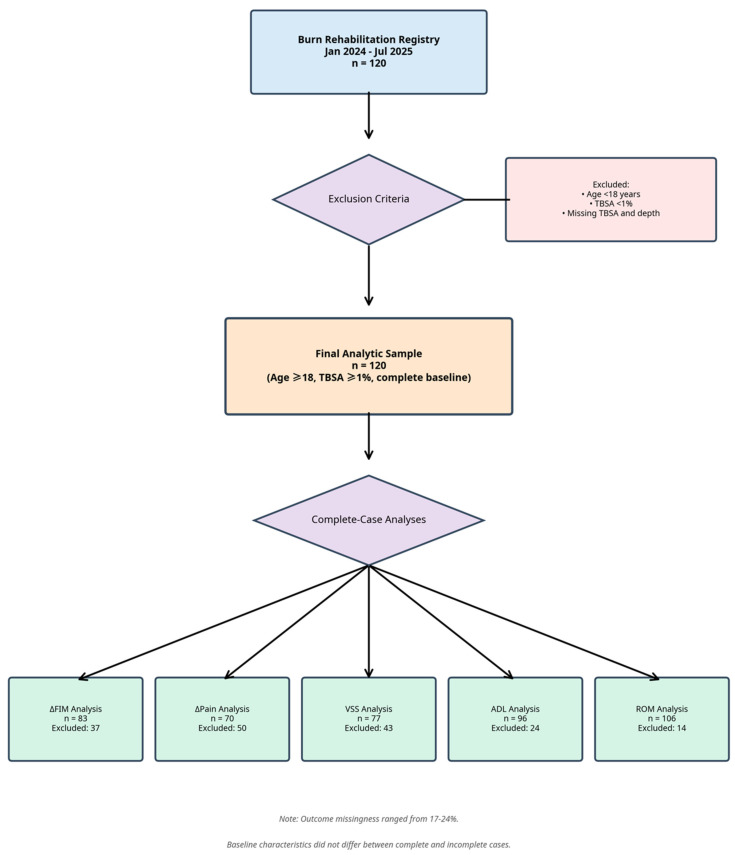
STROBE flow diagram of participant selection. The diagram shows the derivation of the final analytic sample (n = 120) from the Burn Rehabilitation Registry. Due to missing data for outcomes and covariates, multivariable models were conducted on complete-case samples, resulting in analytic sample sizes ranging from n = 70 to n = 92. The potential for selection bias due to missing data was formally assessed and is discussed in Section 2 (see also Supplementary Table S2).

Adults (≥18 years) with confirmed burn injury who received Physical Therapy (PT) or Occupational Therapy (OT) and were entered in the registry between January 2024 and July 2025 were eligible. Patients were excluded if baseline TBSA or burn-depth data were incomplete. The final analytic sample was 120 patients (see Figure 1). Sample size was based on available registry data; no formal sample size calculation was performed.

### 2.3. Data Source and Variables

Data were extracted from electronic health records within the registry. Data completeness and plausibility were checked, and cleaning decisions are documented in Table S1. The research team worked with a de-identified dataset. The cohort was identified using internal registry burn-admission codes. Comorbidities were defined from documented medical record diagnoses.

### 2.4. Predictor Variables

- Patient and Injury Characteristics: Age, gender, ≥1 comorbidity, burn anatomical region, and care setting at initial assessment (Burn ICU/Unit, inpatient ward, outpatient). TBSA was analyzed continuously in regressions and categorized (<10%, 10–19%, 20–39%, ≥40%) for description. Burn depth was classified as superficial, superficial partial thickness, deep partial thickness, full thickness, or mixed.
- Rehabilitation Variables: Therapy duration (days), PT and OT frequency (sessions/week), and provision of scar management, pressure garments, manual therapy, and splinting (each recorded as yes/no). PT sessions/week reflects the frequency of completed PT sessions recorded in the registry, regardless of the specific interventions delivered in each session. In contrast, PT ROM exercises is a separate binary treatment-content indicator capturing whether a structured range-of-motion exercise program was delivered as part of the patient’s PT care (yes/no), independent of the number of PT sessions.
- Care setting: The location of the patient at the time of the initial rehabilitation assessment recorded in the registry (Burn ICU/Unit, inpatient ward, or outpatient). Patients could subsequently transition across settings during their recovery; therefore, the outpatient group may include individuals who previously received inpatient care. Our analyses treat care setting as an index-of-entry setting to reflect initial rehabilitation context rather than the full longitudinal care pathway.

### 2.5. Outcome Measures

- Primary Outcomes: Functional improvement (ΔFIM: admission to discharge change in FIM).
- Secondary Outcomes: Pain reduction (ΔPain: admission to discharge change in Visual Analog Scale (VAS)), scar outcome at follow-up using Vancouver Scar Scale (VSS), full ROM recovery, and achievement of Activity of Daily Living (ADL) improvement goals.
- FIM scores were documented by trained clinical staff at admission and discharge. ADL improvement goals were set by the primary therapist at the start of the rehabilitation episode, and goal attainment was documented at discharge.
- Vancouver Scar Scale (VSS) scores were recorded at follow-up as part of routine registry documentation (i.e., at the end of the rehabilitation episode), rather than immediately at hospital discharge. Because scar maturation evolves over months and is influenced by operative management and time to wound healing, VSS findings should be interpreted in the context of variable follow-up timing across care settings.

### 2.6. Missing Data

Outcome data were missing in 17–24% of patients depending on the measure. All multivariable models were therefore conducted on complete-case samples, with analytic sample sizes ranging from 70 to 92. To assess for potential selection bias, we calculated standardized mean differences (SMDs) for baseline characteristics between patients included and excluded from each multivariable model (Table S2). As several imbalances were noted (|SMD| > 0.25), we acknowledge that data were not missing completely at random (MCAR) and that our results should be interpreted with this potential for bias in mind.

### 2.7. Statistical Analysis

Given the modest sample size (n = 120), we prespecified functional improvement (ΔFIM) as the primary outcome and limited the primary multivariable analysis to one main model. Analyses of pain, scar outcomes, ROM, and ADL were treated as secondary and are presented to describe patterns rather than to make definitive causal inferences.

The analyses were conducted to address the prespecified primary and secondary outcomes described above:

(A) Descriptive Epidemiology: Continuous variables were summarized as mean ± SD or median [IQR]; categorical variables as n (%).
(B) Rehabilitation Frequency Patterns: PT/OT frequency was compared across TBSA, burn depth, and care setting using Mann–Whitney U or Kruskal–Wallis tests.
(C) Association Between Therapy Frequency and Outcomes: One primary multivariable model was fitted for ΔFIM. Four additional models for secondary outcomes (ΔPain, VSS, ROM, and ADL) were conducted as exploratory secondary analyses. Models adjusted for TBSA, burn depth, age, gender, comorbidities, care setting, and baseline scores where relevant. HC3 robust standard errors were used. Collinearity was checked with VIF (problematic if >5); all VIFs were <5 (Table S4).
(D) Association Between Scar Interventions and Scar Outcome: An additional adjusted linear regression examined specific scar interventions vs. VSS.
(E) Service Evaluation: Inpatient vs. outpatient therapy frequency and duration were compared using Mann–Whitney U tests.

Analyses were run in Python 3.11, with *p* < 0.05 considered statistically significant.

### 2.8. Ethical Considerations

IRB approval was obtained. Individual consent was waived because the study was retrospective and used anonymized data.

## 3. Results

### 3.1. Cohort Characteristics and Rehabilitation Received

The final cohort comprised 120 patients (mean age 48.2 ± 17.6 years; 57.5% male) with a median burn size of 18.3% TBSA (IQR 10.6–25.6%) (Table 1). The most prevalent burn depth was superficial partial thickness (35.8%), while mixed-depth burns accounted for 6.7% of injuries (n = 8). Patients received a median of 2.3 PT and 2.7 OT sessions per week, with PT ROM exercises being the most common intervention (70.0%) (Table S5). As expected, therapy frequency was significantly higher for patients with greater TBSA (Figure S1, Table S6) and deeper burns (Table S7), across care settings (Table S8) and for those in inpatient settings compared to outpatients (Figure S2, Table S9).

### 3.2. Predictors of Clinical Outcomes

#### 3.2.1. Primary Outcome: Functional Improvement (ΔFIM)

Patients achieved a mean functional gain (ΔFIM) of 11.3 ± 8.9 points. In the multivariable model (n = 79), initial treatment in the Burn ICU was the only significant predictor of greater functional improvement compared to outpatient care (β = 8.76, *p* = 0.045) (Table S10).

#### 3.2.2. Secondary Outcomes

Pain Reduction (ΔPain): Patients experienced a statistically significant reduction in pain levels from admission to discharge (mean reduction 1.28 points; *p* < 0.001, Table S11). The mean Pain VAS score was 6.38 ± 1.80 at admission (n = 111) and decreased to 5.24 ± 1.99 at discharge (n = 100), with a mean change of 1.28 ± 0.81 (n = 91). In the adjusted model for factors associated with ΔPain (n = 70), none of the included predictors—including therapy frequency, duration, manual therapy, TBSA, burn depth, age, gender, comorbidity status, care setting, or baseline pain—were found to be statistically significant (Table S12).

Scar Severity (VSS): At follow-up, the mean Vancouver Scar Scale (VSS) score for the cohort was 5.0 ± 2.1 (n = 100), indicating mild-to-moderate scarring on average. The adjusted multivariable model (n = 77) revealed three statistically significant predictors of VSS score (Table S13). Having a mixed-depth burn was the strongest predictor, associated with a 2.52-point increase in VSS score compared to other burn depths (95% CI: 0.93 to 4.12, *p* = 0.002). Greater TBSA was also a significant predictor of worse scar outcomes; for every 1% increase in TBSA, the VSS score increased by 0.057 points (95% CI: 0.010 to 0.103, *p* = 0.018). Being treated in an inpatient ward setting was associated with a 1.30-point decrease in VSS score compared to outpatient care (95% CI: −2.39 to −0.20, *p* = 0.020). However, this latter finding is likely an artifact of confounding by indication. The outpatient group had a higher proportion of mixed-depth burns (6.2% vs. 3.5% in the inpatient ward group), which, as the strongest predictor of poor scar outcomes, may explain the paradoxical association. Furthermore, the ICU cohort demonstrates a clear dose–response relationship between injury severity and scar outcome (Table S14), where the most severe injuries (median TBSA 43.4%, 81.8% severe-depth burns) lead to the worst scar outcomes (VSS 7.14 ± 2.07) despite receiving the most intensive therapy (5.4 PT and 6.9 OT sessions/week, 81.8% scar management). This confirms that injury characteristics are the dominant predictors of scar outcome and that care setting acts as a proxy for injury severity rather than an independent causal factor. Notably, after adjusting for all other factors in the model, none of the specific scar management interventions (pressure garments, manual therapy, splinting) or the frequency of therapy (PT or OT sessions per week) were found to be statistically significant predictors of the final VSS score (all *p* > 0.25; Tables S13 and S15).

Activities of Daily Living (ADL): At the end of the observation period, 50.0% of patients (53/106) had achieved their ADL improvement goals. In the adjusted model for ADL improvement (n = 79), longer therapy duration was the only significant predictor, with each additional day of therapy increasing the odds of ADL improvement by 1.4% (OR = 1.014, 95% CI: 1.002 to 1.026, *p* = 0.025) (Table S16).

Range of Motion (ROM): Full ROM recovery was achieved by 56.5% of patients (61/108). Descriptively, recovery rates were lower in patients with greater TBSA severity and in those with mixed-depth burns (Tables S17 and S18). In the corresponding adjusted model (n = 92), having a mixed-depth burn was the only significant predictor, strongly decreasing the odds of achieving full ROM recovery (OR = 0.089, 95% CI: 0.008 to 0.930, *p* = 0.043) (Table S19). This finding aligns with the scar outcome results, further demonstrating that mixed-depth burns represent a particularly challenging injury pattern associated with poorer rehabilitation outcomes across multiple domains. Notably, patients who received specific PT-led ROM exercises had higher rates of full ROM recovery compared to those who did not (Table S20), though this was not a significant predictor in the adjusted model.

## 4. Discussion

This study reports how rehabilitation services were given and what outcomes were achieved in a real group of adult burn survivors. Overall, patients showed clear and clinically meaningful improvement in function and pain. However, long-term outcomes, especially scar quality and range of motion (ROM), were mainly determined by the initial injury, not by how often patients received therapy. This agrees with previous studies that show TBSA, burn depth, and risk of contracture are the main factors that shape recovery [1,2,16]. Mixed-depth burns seemed to be a particularly high-risk pattern, and the links between care setting, therapy dose, and outcomes were strongly affected by differences in baseline severity [6,12].

### 4.1. The Dominance of Injury Severity

Injury severity, especially TBSA and burn depth, was the strongest predictor in all models. This was most clear in the scar analysis, where patients with larger TBSA and mixed-depth burns had worse VSS scores. These findings are similar to previous studies showing that deep and extensive burns, joint involvement, grafting, and delayed healing are major risk factors for hypertrophic scarring, contracture, and disability [2,9,10,16]. Reviews on scar management also report that deep dermal and full-thickness wounds can trigger a strong fibrotic response even when patients receive good-quality care [11,17].

In this cohort, mixed-depth burns predicted both worse scars and failure to regain full ROM. Although only a few studies look directly at this type of burn, risk-factor research supports the idea that heterogeneous tissue damage and long healing time increase the chance of contracture [9,10].

This suggests that mixed-depth burns may represent a distinct clinical phenotype with a higher risk for poor functional and scar outcomes, independent of total burn size. This finding warrants further investigation in larger cohorts to determine whether mixed-depth burns require tailored rehabilitation protocols.

### 4.2. The Nuanced Role of Rehabilitation

It is notable that rehabilitation frequency was not a significant predictor of outcomes in our adjusted models. This null finding does not diminish the importance of rehabilitation but rather suggests that in the acute phase, the profound biological impact of the initial injury characteristics (TBSA, depth) may overwhelm the effect of therapy volume. It is possible that a ‘plateau effect’ exists for session frequency, or that the quality, specific content, and continuity of therapy—variables not captured in our dataset—are more influential than the simple count of sessions per week. Future studies should incorporate more granular measures of rehabilitation delivery to better understand the dose–response relationship in burn recovery.

Reviews of burn rehabilitation indicate that structured exercise and multidisciplinary programs can improve strength, aerobic capacity, and some functional outcomes, yet the evidence for a clear frequency or volume threshold remains weak and heterogeneous [2,13,14,15]. Our lack of a clear independent dose–response signal for weekly sessions is therefore consistent with the current literature.

In contrast, the length of rehabilitation showed an independent association with ADL improvement. This supports guidance that burn rehabilitation often needs to continue for many months, or even years, to keep gains and avoid later decline [2,4,5]. Narrative reviews also stress that staying engaged in regular rehabilitation, rather than relying only on short periods of very intensive therapy, is essential for long-term independence [14,15].

It is also important to note that while pain reduction was statistically significant, the mean discharge pain level remained high at 5.24 on the VAS, with 53% of patients reporting a score >5. This underscores that burn pain is a complex, multifactorial experience that extends beyond the acute phase. From a biopsychosocial perspective, this persistent pain may be influenced by factors not measured in our study, such as neuropathic changes, central sensitization, anxiety, or post-traumatic stress, all of which require a multidisciplinary pain management approach [18,19].

### 4.3. Confounding by Indication: The “Inpatient Advantage” Paradox

Crude analyses showed that ward-based care seemed to give better scar outcomes than outpatient care. However, this difference disappeared after adjusting for case mix: the outpatient group had smaller TBSA but a higher proportion of mixed-depth burns, which strongly predicted poor scars in our model. This pattern reflects confounding by indication rather than a real effect of care setting [6].

The ICU subgroup fits this explanation. Patients treated in the ICU had the largest and deepest burns, received the highest frequency of therapy, and still had the worst VSS scores, showing the well-known link between burn severity and contracture [9,10]. Care settings are arranged by increasing injury severity. Despite stepwise increases in therapy frequency, scar outcomes progressively worsened from outpatient to ICU, demonstrating that injury severity dominates rehabilitation effects. This frequency–response relationship validates the confounding by indication observed in the regression model, where care setting acts as a proxy for injury severity. Descriptions of burn care pathways also note that ICU and ward care mainly reflect how severe the injury is, with rehabilitation delivered across all settings [5,12]. Our findings therefore stress that careful risk adjustment is essential when comparing outcomes between different services.

### 4.4. Strengths and Limitations

The strengths of this study include the use of real-world registry data, a wide range of outcomes (function, pain, ROM, ADL, and scars), and multivariable models that adjust for the main confounders. The choice of FIM and VSS is supported by earlier burn-specific psychometric studies, although both tools have known limits, such as reduced responsiveness and ceiling effects [6,20,21].

This study has several limitations. First, the study’s modest sample size (n = 120), and particularly the small subgroup of patients with mixed-depth burns (n = 8, 6.7%), limits the statistical power of our analyses. This is reflected in the wide confidence intervals for some predictors, such as for mixed-depth burns in the ROM recovery model (OR 0.089, 95% CI: 0.008–0.930). Consequently, while our findings regarding mixed-depth burns as a potentially high-risk phenotype are statistically significant, they should be interpreted as hypothesis-generating and require validation in larger, multi-center cohorts.

Second, our measurement of rehabilitation ‘dose’ as sessions per week is a simplification that does not capture the specific content, intensity, or duration of each therapy session. Furthermore, our models could not adjust for several important unmeasured confounders, such as surgical grafting, wound healing time, the variable time interval of VSS follow-up, the involvement of critical joints (e.g., hands, face), specific pain management protocols, or psychosocial factors, all of which could influence recovery. We did not have complete data on operative procedures (e.g., grafting/number of operations), mechanism of injury, or time to wound healing; these factors likely influence scarring and ROM recovery and may contribute to residual confounding.

Third, the study’s single-center, retrospective design may limit the generalizability of our findings. The reliance on complete-case analysis, as discussed, also introduces a potential for bias. These issues limit causal inference and may partly explain the absence of a clear independent effect of therapy frequency. Future multi-center prospective studies are needed to validate these results and further explore the impact of mixed-depth burns on long-term recovery.

### 4.5. Clinical Implications and Future Directions

Clinically, mixed-depth burns should be seen as a high-risk pattern for adverse scar and ROM outcomes, in line with risk-factor reviews on deep and joint-involving burns [9,10,11]. These patients may need early identification, close follow-up, and more intensive or innovative strategies to protect joint movement and improve scar quality.

The observed link between longer therapy frequency and ADL improvement indicates that health systems should prioritize care models that provide ongoing access to rehabilitation after the acute admission, consistent with guideline advice for early and continuing rehabilitation [2,4,5,7].

Future research should use prospective, multi-center cohorts and collect more detailed rehabilitation data, including timing, content, and adherence, to better describe dose–response relationships [6,8,13,14]. In addition, studies that focus specifically on mixed-depth burns, using modern scar scales and patient-reported outcome measures, are needed to test targeted rehabilitation strategies.

## 5. Conclusions

In summary, the findings support earlier evidence that the severity and type of the initial burn injury are the main factors shaping long-term physical outcomes [1,2]. Rehabilitation is still a crucial part of care, but in observational data, its measurable effects are closely linked to the underlying injury characteristics [6,15]. Mixed-depth burns were a strong predictor of poor scar and ROM outcomes, so this patient group needs special attention and further research [9,10]. Improving risk stratification and building stronger evidence on the dose and content of rehabilitation will be important for optimizing long-term outcomes in burn survivors [13,14].

## Figures and Tables

**Table 1:** Cohort characteristics (n = 120).

Characteristic	Value
Age (years), mean ± SD	48.2 ± 17.6
Sex, n (%)	
Male	69 (57.5)
Female	51 (42.5)
Has comorbidity, n (%)	71 (59.2)
TBSA (%), median [IQR]	18.3 [10.6–25.6]
TBSA severity group, n (%)	
<10%	27 (22.5)
10–19%	40 (33.3)
20–39%	33 (27.5)
≥40%	20 (16.7)
Burn depth, n (%)	
Superficial	11 (9.2)
Superficial partial thickness	43 (35.8)
Deep partial thickness	32 (26.7)
Full thickness	26 (21.7)
Mixed-depth	8 (6.7)
Care setting, n (%)	
Burn ICU/Unit	14 (11.7)
Inpatient ward	67 (55.8)
Outpatient	39 (32.5)
PT ROM exercises, n (%)	84 (70.0)
Scar management, n (%)	56 (46.7)
Pressure garments, n (%)	40 (33.3)
Manual therapy, n (%)	47 (39.2)
Splinting, n (%)	34 (28.3)

n = 120 patients included in analysis. Continuous variables presented as mean ± SD or median [IQR] depending on distribution. Categorical variables presented as n (%).

## Data Availability

The dataset and analysis code supporting the conclusions of this article are included within the article’s additional files.

## Supplementary Materials

The following supporting information can be downloaded at https://www.mdpi.com/article/10.3390/ebj7010006/s1, Table S1. Data cleaning log; Table S2. Standardized Mean Differences (SMDs) Between Patients Included vs. Excluded from Each Regression Model; Table S3. Missingness summary; Table S4. Variance inflation factors (VIF) for multivariable models; Table S5. Provision of PT-led ROM exercises by burn depth; Table S6. Rehabilitation frequency and episode duration by TBSA severity; Table S7. Rehabilitation frequency by burn depth; Table S8. Rehabilitation frequency and episode duration by care setting; Table S9. Service evaluation: inpatient vs. outpatient rehabilitation; Table S10. Adjusted model for functional improvement (ΔFIM); Table S11. Pain outcomes (VAS) at admission and discharge; Table S12. Adjusted model for pain reduction (ΔPain); Table S13. Adjusted model for scar outcome (VSS); Table S14. Severity, rehabilitation volume, and scar outcomes by care setting; Table S15. Scar management components among patients receiving scar care; Table S16. Adjusted model for ADL improvement; Table S17. ROM improvement by TBSA severity; Table S18. ROM improvement by burn depth; Table S19. Adjusted model for full ROM recovery; Table S20. Provision of PT-led ROM exercises by TBSA severity; Figure S1. Distribution of total body surface area burned (TBSA); Figure S2. Distribution of Burn Depth Categories; Figure S3. Rehabilitation Intervention Rates; Figure S4. Rehabilitation dose by TBSA severity; Figure S5. Inpatient vs. Outpatient Service Comparison.

## Abbreviations

The following abbreviations are used in this manuscript:

TBSA	Total Body Surface Area
FIM	Functional Independence Measure
VSS	Vancouver Scar Scale
VAS	Visual Analog Scale
ADL	Activities of Daily Living
ROM	Range of Motion
PT	Physical Therapy
OT	Occupational Therapy
ICU	Intensive Care Unit
