# Supplementary material for: Association Between Rehabilitation Frequency and Functional Outcomes After Burn Injury: A Single-Center Retrospective Analysis of Confounding by Indication
Source: Eur Burn J. 2026 Jan 19;7(1):6. doi: 10.3390/ebj7010006 (PMC12922151; doi:10.3390/ebj7010006)
Supplement: Supplementary file 1 [file ebj-07-00006-s001.zip › ebj-4053462-supplementary.docx]

**Table S1. Data cleaning log.**

| **Variable** | **Issue identified** | **Action taken** | **N affected** |
| --- | --- | --- | --- |
| Duration_days | Missing end date | Recoded duration as missing | 2 |
| Pain_VAS_discharge | Discharge value > admission value (illogical) | Recoded discharge pain as missing | 4 |
| FIM_discharge | Discharge value < admission value (illogical) | Recoded discharge FIM as missing | 12 |

n To assess for potential selection bias, we calculated standardized mean differences (SMDs) for baseline characteristics between patients included and excluded from each multivariable model (Supplementary Table S2). As several imbalances were noted (|SMD| > 0.25), we acknowledge that missingness was not completely at random.

**Table S2: Standardized Mean Differences (SMDs) Between Patients Included vs. Excluded from Each Regression Model.**

| **Characteristic** | **ΔFIM (n=79/41)** | **ΔPain (n=70/50)** | **VSS (n=77/43)** | **ROM (n=92/28)** | **ADL (n=79/41)** |
| --- | --- | --- | --- | --- | --- |
| Age (years) | 0.30 | -0.20 | -0.28 | 0.09 | 0.01 |
| TBSA (%) | 0.01 | 0.00 | 0.12 | 0.13 | 0.00 |
| Gender (Male) | 0.17 | -0.31 | 0.12 | 0.25 | 0.01 |
| Has comorbidity | 0.06 | -0.33 | 0.09 | -0.06 | 0.06 |
| PT sessions/week | -0.24 | -0.19 | 0.12 | 0.16 | -0.22 |
| OT sessions/week | 0.02 | 0.37 | -0.10 | -0.03 | -0.07 |
| Therapy duration | 0.11 | -0.02 | -0.15 | -0.10 | 0.11 |

SMD = Standardized Mean Difference. Complete = patients included in the multivariable regression model; Incomplete = patients excluded due to missing outcome or covariate data. Meaningful imbalance is often defined as |SMD| > 0.25.

**Table S3. Missingness summary.**

| **Variable** | **N missing** | **% missing** |
| --- | --- | --- |
| Scar_management_type | 67 | 56% |
| Pain_VAS_discharge | 20 | 17% |
| VSS_followup | 20 | 17% |
| ROM_improvement_deg | 17 | 14% |
| FIM_discharge | 14 | 12% |
| PT_sessions_per_week | 14 | 12% |
| ADL_improvement_yesno | 14 | 12% |
| ROM_full_flag | 12 | 10% |
| OT_sessions_per_week | 10 | 8% |
| Pain_VAS_admission | 9 | 8% |
| FIM_admission | 6 | 5% |
| PT_ROM_exercises_yesno | 4 | 3% |
| Therapy_end_date | 2 | 2% |
| Therapy_duration_days | 2 | 2% |
| Anatomical_region | 0 | 0% |
| Burn_depth_cat | 0 | 0% |
| Therapy_start_date | 0 | 0% |
| TBSA_group | 0 | 0% |
| TBSA_percent | 0 | 0% |
| Comorbidity_count | 0 | 0% |
| Has_comorbidity | 0 | 0% |
| Patient_ID | 0 | 0% |
| Age_years | 0 | 0% |
| Gender | 0 | 0% |
| Care_setting | 0 | 0% |
| Splinting_yesno | 0 | 0% |
| Manual_therapy_yesno | 0 | 0% |
| Pressure_garments_yesno | 0 | 0% |
| Scar_management_yesno | 0 | 0% |

n=120 patients. Percentage of missing values for each variable in the dataset.

**Table S4. Variance inflation factors (VIF) for multivariable models.**

| **Variable** | **VIF** |
| --- | --- |
| TBSA_percent | 4.55 |
| PT_sessions_per_week | 3.35 |
| OT_sessions_per_week | 3.46 |
| Therapy_duration_days | 2.42 |
| Age_years | 4.40 |

Variance Inflation Factor (VIF) values for predictor variables in multivariable models. VIF <5 indicates acceptable collinearity; VIF <10 is generally considered acceptable.

**Table S5. Provision of PT-led ROM exercises by burn depth.**

| **Burn depth** | **N total** | **N with PT ROM exercises** | **%** |
| --- | --- | --- | --- |
| Superficial | 11 | 6 | 55% |
| Superficial partial thickness | 43 | 29 | 67% |
| Deep partial thickness | 32 | 24 | 75% |
| Full thickness | 26 | 19 | 73% |
| Mixed depth | 8 | 6 | 75% |

N=120 patients. Frequency of structured PT ROM exercises documented (yes/no) across burn depth categories. Values presented as n (%).

**Table S6. Rehabilitation frequency and episode duration by TBSA severity.**

| **TBSA group** | **N** | **PT sessions/week, median [IQR]** | **OT sessions/week, median [IQR]** | **Duration (days), median [IQR]** |
| --- | --- | --- | --- | --- |
| <10% | 27 | 1.1 [0.5–2.2] | 2.1 [1.4–2.6] | 67 [41–80] |
| 10–19% | 40 | 1.8 [1.1–3.7] | 1.9 [1.0–3.7] | 59 [38–71] |
| 20–39% | 33 | 3.1 [1.6–4.5] | 4.7 [1.8–6.4] | 68 [37–140] |
| ≥40% | 20 | 4.3 [3.0–7.0] | 6.6 [2.8–7.0] | 46 [27–84] |
| **Kruskal–Wallis p-value** |  | **0.0001** | **0.0002** | 0.3455 |

n=120 patients. Values presented as median [IQR]. P-values from Kruskal–Wallis test comparing across TBSA groups.

**Table S7. Rehabilitation frequency by burn depth.**

| **Burn depth** | **N** | **PT sessions/week, median [IQR]** | **OT sessions/week, median [IQR]** |
| --- | --- | --- | --- |
| Superficial | 11 | 1.2 [0.5–2.0] | 1.4 [0.6–1.7] |
| Superficial partial thickness | 43 | 1.6 [0.9–3.0] | 2.0 [1.0–3.4] |
| Deep partial thickness | 32 | 2.4 [1.1–3.7] | 3.5 [1.8–6.1] |
| Full thickness | 26 | 4.5 [2.8–7.0] | 5.6 [2.7–7.0] |
| Mixed depth | 8 | 3.3 [1.4–4.3] | 4.1 [3.3–5.1] |

n=120 patients. Values presented as median [IQR]. P-values from Kruskal–Wallis test comparing across depth categories.

**Table S8. Rehabilitation frequency and episode duration by care setting.**

| **Care setting** | **N** | **PT sessions/week, median [IQR]** | **OT sessions/week, median [IQR]** | **Duration (days), median [IQR]** |
| --- | --- | --- | --- | --- |
| Burn ICU/Unit | 14 | 6.2 [3.4–7.0] | 7.0 [4.9–7.0] | 74 [30–90] |
| Inpatient ward | 67 | 2.9 [1.2–4.1] | 3.0 [1.8–5.8] | 48 [30–68] |
| Outpatient | 39 | 1.2 [0.8–2.3] | 1.6 [0.9–2.7] | 89 [58–165] |

n=120 patients. Values presented as median [IQR]. P-values from Mann–Whitney U test comparing inpatient (ICU + Ward combined) vs. outpatient.

**Table S9. Service evaluation: inpatient vs. outpatient rehabilitation.**

| **Variable** | **Inpatient, median [IQR]** | **Outpatient, median [IQR]** | **Test** | **P-value** |
| --- | --- | --- | --- | --- |
| Therapy duration (days) | 51 [30–70] | 89 [58–165] | Mann–Whitney U | **<0.001** |
| PT sessions/week | 3.3 [1.3–6.2] | 1.2 [0.8–2.3] | Mann–Whitney U | **0.0004** |
| OT sessions/week | 3.4 [1.8–7.0] | 1.6 [0.9–2.7] | Mann–Whitney U | **<0.001** |

n=120 patients (Inpatient n=81, Outpatient n=39). Values presented as median [IQR] or n (%). P-values from Mann–Whitney U test (continuous) or chi-square test (categorical).

**Table S10. Adjusted model for functional improvement (ΔFIM).**

| **Predictor** | **Beta** | **95% CI** | **P-value** |
| --- | --- | --- | --- |
| PT_sessions_per_week | −0.258 | −1.434 to 0.919 | 0.668 |
| OT_sessions_per_week | 0.388 | −0.672 to 1.449 | 0.473 |
| Therapy_duration_days | 0.013 | −0.034 to 0.060 | 0.577 |
| PT_ROM_exercises | −0.531 | −5.652 to 4.591 | 0.839 |
| TBSA_percent | −0.026 | −0.306 to 0.253 | 0.854 |
| Depth_Superficial | −2.79 | −11.781 to 6.201 | 0.543 |
| Depth_Deep_partial_thickness | 5.026 | −0.811 to 10.863 | 0.092 |
| Depth_Full_thickness | 3.617 | −3.694 to 10.929 | 0.332 |
| Depth_Mixed_depth | 1.173 | −7.114 to 9.461 | 0.781 |
| Age_years | 0.048 | −0.096 to 0.193 | 0.513 |
| Gender_Male | 3.675 | −0.484 to 7.833 | 0.083 |
| Has_comorbidity_Yes | −1.822 | −6.294 to 2.649 | 0.424 |
| Setting_ICU | 8.761 | 0.202 to 17.320 | **0.045** |
| Setting_Inpatient | 0.694 | −5.291 to 6.679 | 0.820 |
| FIM_admission | 0.145 | −0.139 to 0.429 | 0.317 |

n=79 patients with complete data for ΔFIM and all covariates. A multivariable linear regression model with robust standard errors was used, adjusting for TBSA group, burn depth, age, gender, comorbidity status, care setting, and baseline FIM score. β = regression coefficient; CI = confidence interval.

**Table S11. Pain outcomes (VAS) at admission and discharge.**

| **Outcome** | **N** | **Mean ± SD** | **Median [IQR]** |
| --- | --- | --- | --- |
| Pain VAS at admission | 111 | 6.38 ± 1.80 | 6.3 [5.1–7.5] |
| Pain VAS at discharge | 100 | 5.24 ± 1.99 | 5.2 [3.7–6.7] |
| Pain reduction (ΔPain) | 91 | 1.28 ± 0.81 | 1.1 [0.6–1.9] |

N=120 patients. VAS scores range 0-10. Values presented as mean ± SD. Ns vary due to missing data at each timepoint. Pain reduction (ΔPain) was statistically significant (p < 0.001, paired t-test).

**Table S12. Adjusted model for pain reduction (ΔPain).**

| **Predictor** | **Beta** | **95% CI** | **P-value** |
| --- | --- | --- | --- |
| PT_sessions_per_week | 0.053 | −0.078 to 0.184 | 0.426 |
| OT_sessions_per_week | 0.048 | −0.079 to 0.176 | 0.458 |
| Therapy_duration_days | −0.002 | −0.007 to 0.003 | 0.501 |
| Manual_therapy | 0.060 | −0.440 to 0.560 | 0.816 |
| TBSA_percent | −0.004 | −0.026 to 0.019 | 0.746 |
| Depth_Superficial | 0.193 | −0.824 to 1.211 | 0.709 |
| Depth_Deep_partial_thickness | −0.043 | −0.663 to 0.577 | 0.893 |
| Depth_Full_thickness | 0.376 | −0.429 to 1.181 | 0.360 |
| Depth_Mixed_depth | −0.174 | −1.165 to 0.817 | 0.731 |
| Age_years | 0.006 | −0.009 to 0.022 | 0.436 |
| Gender_Male | −0.226 | −0.760 to 0.308 | 0.408 |
| Has_comorbidity_Yes | −0.207 | −0.671 to 0.257 | 0.382 |
| Setting_ICU | −0.326 | −1.360 to 0.707 | 0.536 |
| Setting_Inpatient | −0.144 | −0.822 to 0.533 | 0.677 |
| Pain_VAS_admission | −0.023 | −0.248 to 0.202 | 0.841 |

n = 70 patients with complete data for **ΔPain** and all covariates. A multivariable linear regression model with robust standard errors was used, adjusting for TBSA group, burn depth, age, gender, comorbidity status, care setting, and baseline Pain VAS. **β** = regression coefficient; **CI** = confidence interval.

**Table S13. Adjusted model for scar outcome (VSS).**

| **Predictor** | **Beta** | **95% CI** | **P-value** |
| --- | --- | --- | --- |
| Scar_management | 0.106 | −0.931 to 1.143 | 0.841 |
| Pressure_garments | −0.358 | −1.367 to 0.651 | 0.486 |
| Manual_therapy | −0.491 | −1.567 to 0.586 | 0.372 |
| Splinting | −0.650 | −1.781 to 0.481 | 0.260 |
| PT_sessions_per_week | 0.008 | −0.243 to 0.259 | 0.948 |
| OT_sessions_per_week | 0.179 | −0.049 to 0.406 | 0.124 |
| Therapy_duration_days | −0.009 | −0.019 to 0.001 | 0.082 |
| TBSA_percent | 0.057 | 0.010 to 0.103 | **0.018** |
| Depth_Superficial | 1.027 | −0.822 to 2.876 | 0.276 |
| Depth_Deep_partial_thickness | 0.407 | −0.819 to 1.634 | 0.515 |
| Depth_Full_thickness | 1.293 | −0.126 to 2.711 | 0.074 |
| Depth_Mixed_depth | 2.523 | 0.927 to 4.118 | **0.002** |
| Age_years | −0.013 | −0.040 to 0.014 | 0.355 |
| Gender_Male | −0.607 | −1.664 to 0.451 | 0.261 |
| Has_comorbidity_Yes | 0.623 | −0.524 to 1.770 | 0.287 |
| Setting_ICU | −0.838 | −3.595 to 1.918 | 0.551 |
| Setting_Inpatient | −1.298 | −2.392 to −0.203 | **0.020** |

n = 77 patients with complete data for **VSS** and all covariates. A multivariable linear regression model with robust standard errors was used, adjusting for TBSA group, burn depth, age, gender, comorbidity status, care setting, therapy duration, and scar-management variables. **β** = regression coefficient; **CI** = confidence interval.

**Table S14. Severity, rehabilitation volume, and scar outcomes by care setting.**

| **Characteristic** | **Outpatient (n = 32)** | **Inpatient ward (n = 57)** | **ICU (n = 11)** |
| --- | --- | --- | --- |
| TBSA (%), median | 15.8 | 17.8 | 43.4 |
| Severe-depth burns, n (%) | 4 (12.5%) | 15 (26.3%) | 9 (81.8%) |
| Therapy volume (PT+OT sessions/week), median | 3.2 | 6.2 | 12.3 |
| VSS score, mean | 4.73 | 4.74 | 7.14 |

Data are presented for the subset of patients with complete VSS data (n=77).

**Table S15. Scar management components among patients receiving scar care.**

| **Component** | **N** | **% of scar-managed patients (n = 56)** |
| --- | --- | --- |
| Stretching/ROM program | 39 | 70% |
| Pressure garment | 37 | 66% |
| Silicone/gel sheet | 31 | 55% |
| Moisturizer/massage | 31 | 55% |
| Manual scar mobilization | 18 | 32% |
| Splinting/positioning | 15 | 27% |

n=56 patients who received scar management (Scar_management_yesno = Yes). Frequencies of specific scar management techniques used. Patients could receive multiple interventions. For the association between scar management interventions and VSS outcome, see the adjusted regression model in Table S13.

**Table S16. Adjusted model for ADL improvement.**

| **Predictor** | **OR** | **95% CI** | **P-value** |
| --- | --- | --- | --- |
| PT_sessions_per_week | 0.931 | 0.725 to 1.197 | 0.579 |
| OT_sessions_per_week | 1.026 | 0.818 to 1.287 | 0.823 |
| Therapy_duration_days | 1.014 | 1.002 to 1.026 | **0.025** |
| PT_ROM_exercises | 0.875 | 0.324 to 2.359 | 0.792 |
| TBSA_percent | 1.011 | 0.974 to 1.050 | 0.551 |
| Depth_Superficial | 0.171 | 0.017 to 1.766 | 0.138 |
| Depth_Deep_partial_thickness | 0.723 | 0.217 to 2.409 | 0.597 |
| Depth_Full_thickness | 0.769 | 0.190 to 3.103 | 0.712 |
| Depth_Mixed_depth | 0.382 | 0.047 to 3.122 | 0.369 |
| Age_years | 0.996 | 0.968 to 1.024 | 0.758 |
| Gender_Male | 0.432 | 0.164 to 1.136 | 0.089 |
| Has_comorbidity_Yes | 1.557 | 0.556 to 4.355 | 0.399 |
| Setting_ICU | 2.185 | 0.279 to 17.116 | 0.457 |
| Setting_Inpatient | 3.158 | 0.736 to 13.555 | 0.122 |

n=79 patients with complete data for ADL improvement and all covariates. Logistic regression adjusting for TBSA group, burn depth, age, gender, comorbidity status, care setting, and duration. OR = odds ratio; CI = confidence interval.

**Table S17. ROM improvement by TBSA severity.**

| **TBSA group** | **N** | **ROM improvement (deg), mean ± SD** | **Median [IQR]** |
| --- | --- | --- | --- |
| <10% | 9 | 33.4 ± 18.8 | 32.0 [23.0–36.0] |
| 10–19% | 13 | 31.7 ± 17.2 | 32.0 [25.0–39.0] |
| 20–39% | 10 | 43.1 ± 15.4 | 43.0 [37.0–52.2] |
| ≥40% | 8 | 48.9 ± 15.3 | 53.0 [35.8–56.2] |

n=44 patients. ROM improvement in degrees among patients without full ROM at baseline. Values presented as mean ± SD. P-value from Kruskal–Wallis test.

**Table S18. ROM improvement by burn depth.**

| **Burn depth** | **N** | **ROM improvement (deg), mean ± SD** | **Median [IQR]** |
| --- | --- | --- | --- |
| Superficial | 2 | 19.5 ± 20.5 | 19.5 [12.2–26.8] |
| Superficial partial thickness | 12 | 28.0 ± 12.3 | 32.0 [22.5–34.5] |
| Deep partial thickness | 14 | 42.6 ± 17.2 | 37.5 [30.0–51.5] |
| Full thickness | 6 | 52.2 ± 20.3 | 57.0 [39.0–67.5] |
| Mixed depth | 6 | 41.8 ± 11.1 | 42.0 [33.2–50.8] |

ROM improvement by burn depth for the subset of patients who had not achieved full ROM at admission and had complete follow-up data (n=44).

**Table S19. Adjusted model for full ROM recovery.**

| **Predictor** | **OR** | **95% CI** | **P-value** |
| --- | --- | --- | --- |
| PT_sessions_per_week | 0.982 | 0.782 to 1.234 | 0.878 |
| PT_ROM_exercises | 1.423 | 0.580 to 3.489 | 0.441 |
| Splinting | 0.542 | 0.213 to 1.381 | 0.199 |
| TBSA_percent | 1.014 | 0.978 to 1.051 | 0.451 |
| Depth_Superficial | 1.060 | 0.221 to 5.081 | 0.942 |
| Depth_Deep_partial_thickness | 0.549 | 0.184 to 1.636 | 0.282 |
| Depth_Full_thickness | 0.364 | 0.101 to 1.315 | 0.123 |
| Depth_Mixed_depth | 0.089 | 0.008 to 0.930 | **0.043** |
| Age_years | 0.988 | 0.963 to 1.014 | 0.373 |
| Gender_Male | 1.249 | 0.522 to 2.991 | 0.617 |
| Has_comorbidity_Yes | 1.180 | 0.477 to 2.924 | 0.720 |
| Setting_ICU | 1.174 | 0.204 to 6.744 | 0.857 |
| Setting_Inpatient | 0.870 | 0.331 to 2.287 | 0.778 |

n=92 patients with complete data for ROM full status and all covariates. Logistic regression adjusting for TBSA group, burn depth, age, gender, comorbidity status, care setting, duration, and PT ROM exercises. OR = odds ratio; CI = confidence interval.

**Table S20. Provision of PT-led ROM exercises by TBSA severity.**

| **TBSA group** | **N total** | **N with PT ROM exercises** | **%** |
| --- | --- | --- | --- |
| <10% | 27 | 16 | 59% |
| 10–19% | 38 | 28 | 74% |
| 20–39% | 32 | 23 | 72% |
| ≥40% | 19 | 17 | 89% |

Provision of PT-led ROM exercises by TBSA severity group (n=116). There was no statistically significant association between TBSA group and the provision of PT ROM exercises (Chi-square test, p = 0.162).


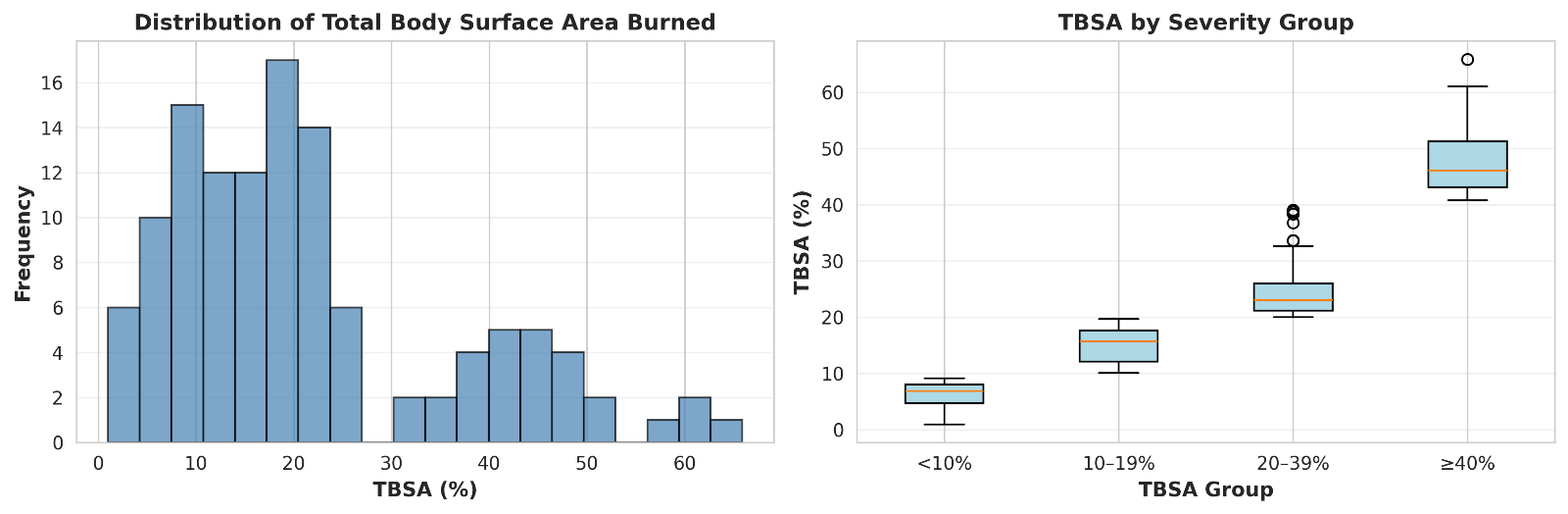


**Figure S1.** Distribution of total body surface area burned (TBSA). Left panel: Histogram showing the frequency distribution of TBSA (%) across the 120 patients. Right panel: Boxplots of TBSA (%) by severity group (<10%, 10–19%, 20–39%, ≥40%). Median TBSA was 17.4% (IQR 10.0–29.8). Abbreviations: TBSA, total body surface area; IQR, interquartile range.


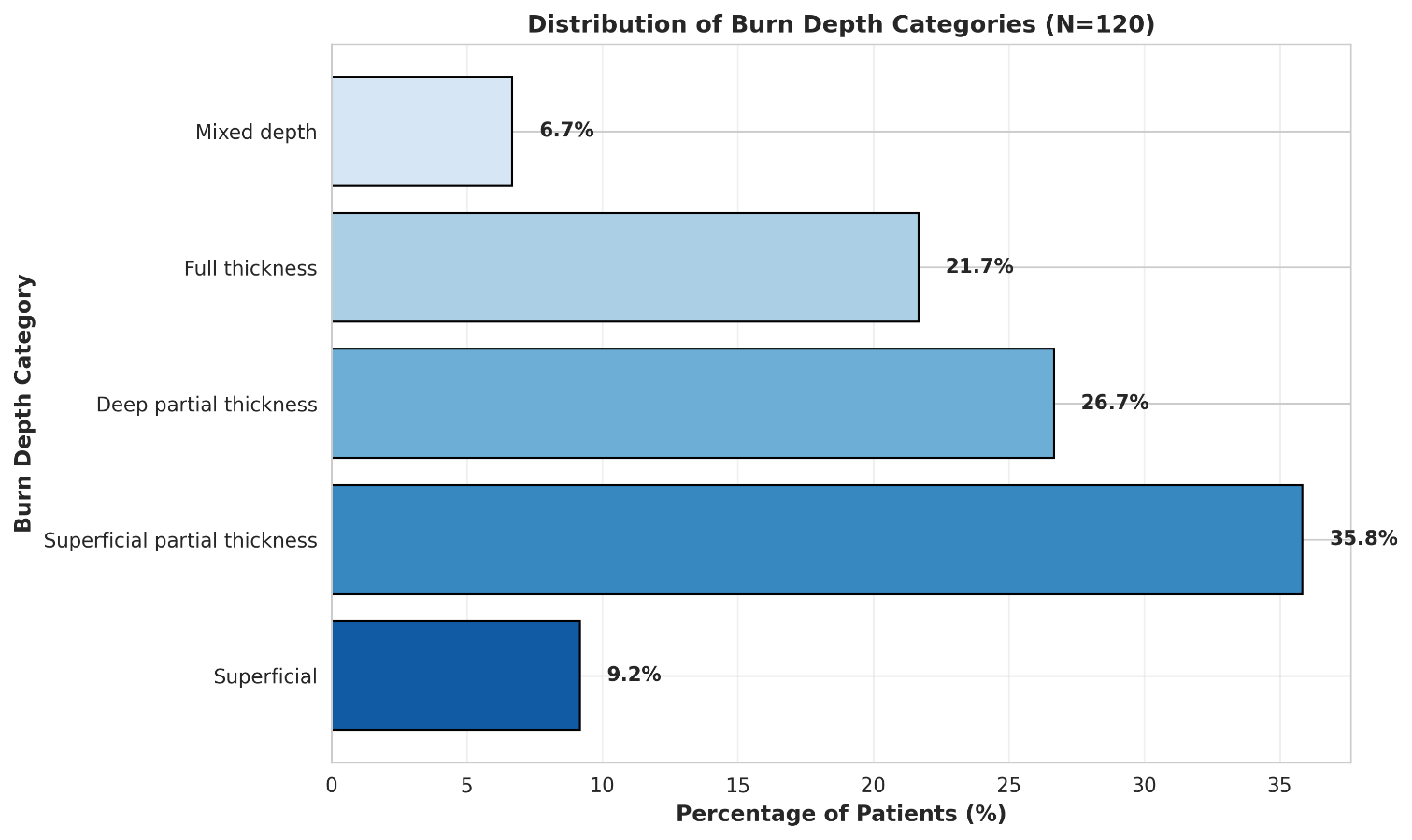


**Figure S2. Distribution of Burn Depth Categories.** Horizontal bar chart showing the percentage of patients in each burn depth category (N=120). Superficial partial thickness burns were the most common (43.3%), followed by deep partial thickness (25.0%), full thickness (14.2%), superficial (9.2%), and mixed depth (8.3%). Percentages are displayed at the end of each bar.


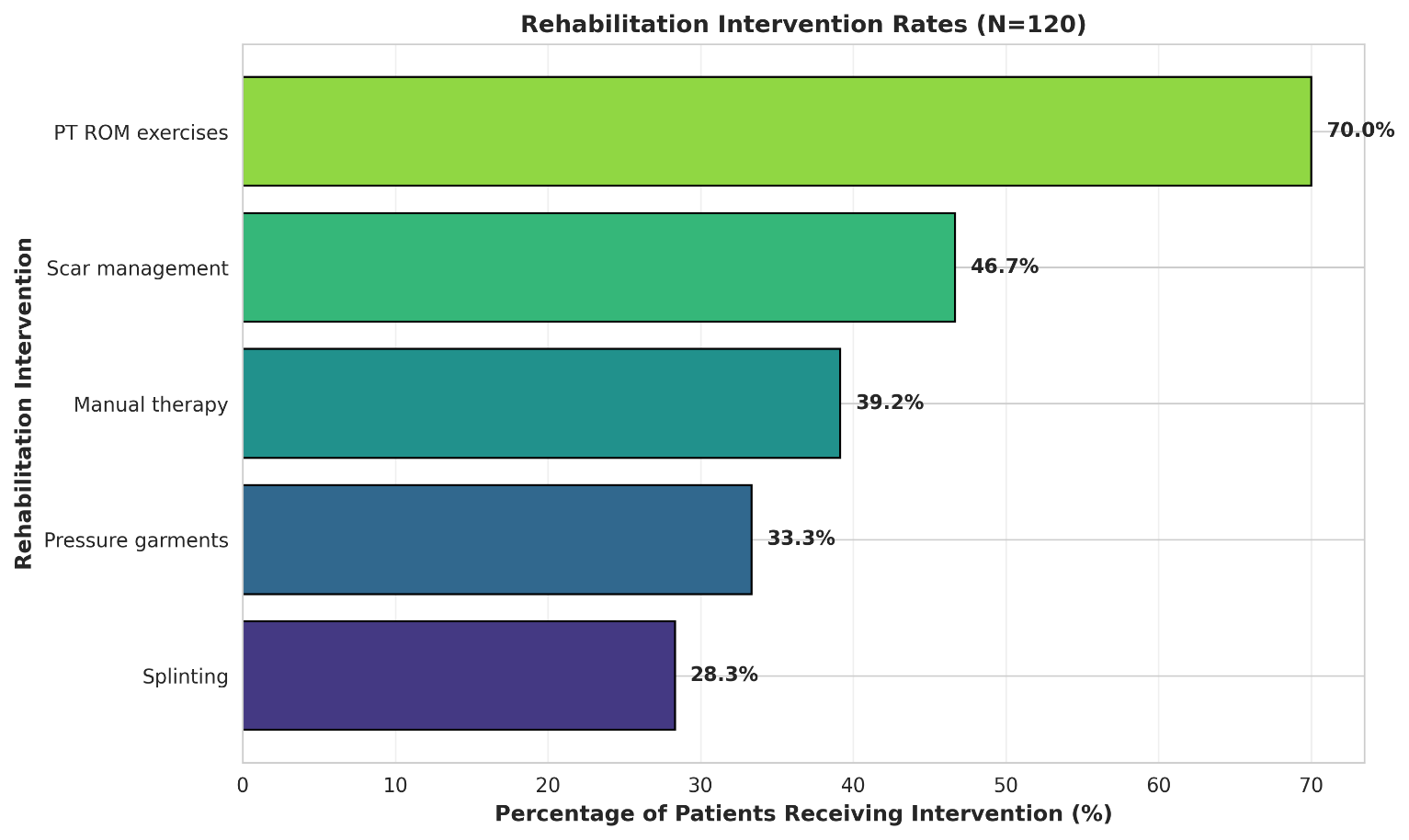


**Figure S3. Rehabilitation Intervention Rates.** Horizontal bar chart displaying the percentage of patients receiving each rehabilitation intervention (N=120). PT ROM exercises were the most common intervention (70.0%), followed by scar management (46.7%), pressure garments (40.0%), manual therapy (34.2%), and splinting (30.0%). Percentages are displayed at the end of each bar. PT = physical therapy; ROM = range of motion.


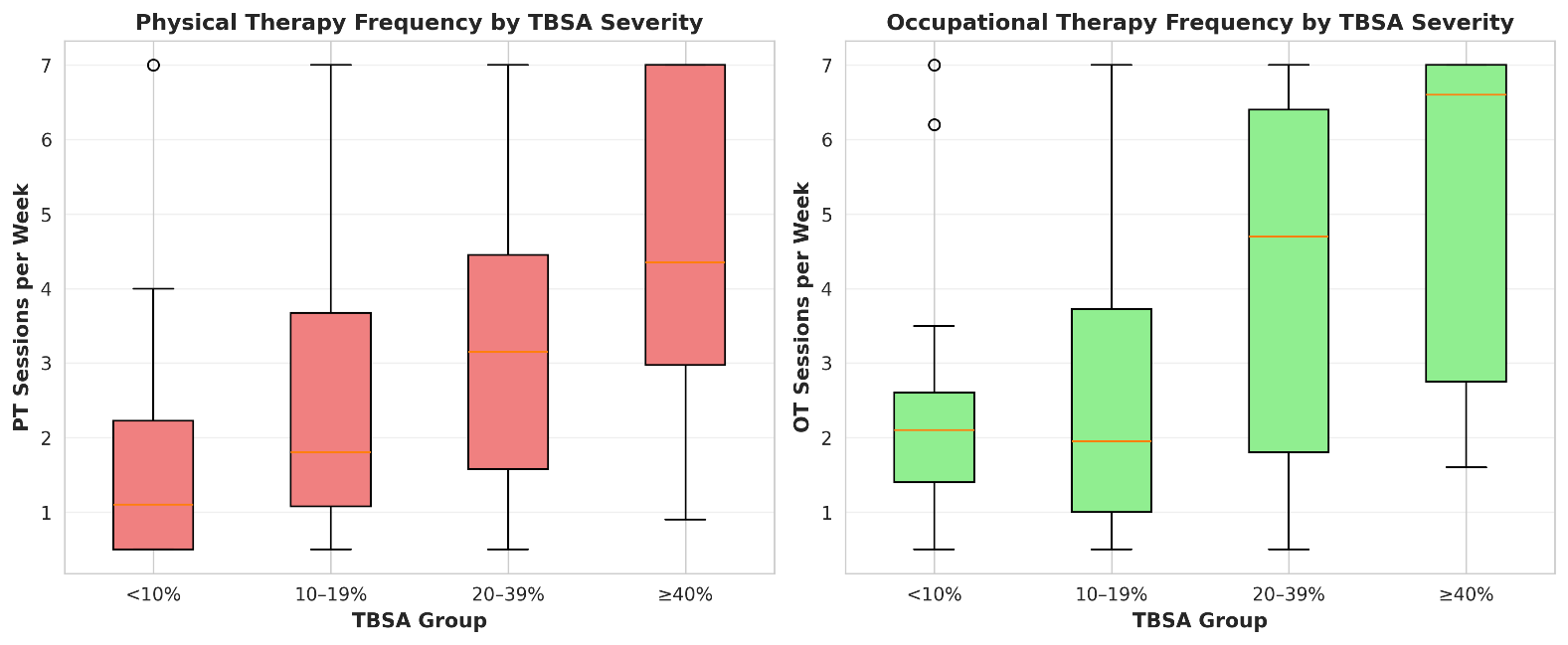


**Figure S4. Rehabilitation dose by TBSA severity.** Left panel: Boxplots of PT sessions per week by TBSA severity group. Median PT dose increased from 1.5 sessions/week in the <10% TBSA group to 3.0 sessions/week in the ≥40% TBSA group (Kruskal–Wallis p = 0.005). Right panel: Boxplots of OT sessions per week by TBSA severity group, showing a similar increasing trend (Kruskal–Wallis p = 0.002). Abbreviations: PT, physical therapy; OT, occupational therapy; TBSA, total body surface area.


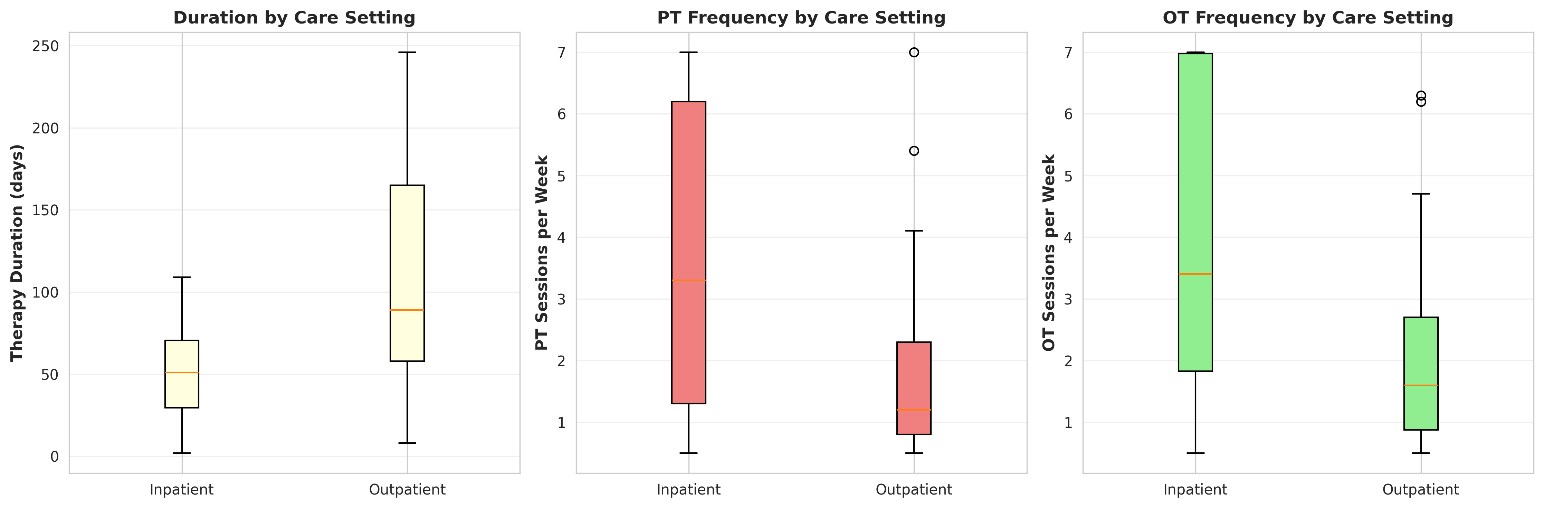


**Figure S5. Inpatient vs. Outpatient Service Comparison.** Three boxplots comparing rehabilitation service characteristics between inpatient (Burn ICU/Unit + Inpatient ward, n=90) and outpatient (n=30) settings. Left panel: Therapy duration in days (median 55 vs. 102 days, p=0.001). Middle panel: PT sessions per week (median 2.5 vs. 1.5, p=0.038). Right panel: OT sessions per week (median 3.0 vs. 1.5, p<0.001). All comparisons used Mann–Whitney U tests. PT = physical therapy; OT = occupational therapy.
